# Effect of Intravenous Glutamine on *Caspase-12* Expression in the Apoptosis of the Glomerular Epithelial Cells of Male Rats Exposed to Cisplatin

**DOI:** 10.31557/APJCP.2021.22.2.457

**Published:** 2021-02

**Authors:** Tamara Aulia Fakhrinnisa, Imam Susilo, Arifa Mustika, Miyayu Soneta Sofyan

**Affiliations:** 1 *Medicine Undergraduate Program, Faculty of Medicine, Airlangga University, Jalan Mayjen Prof. Dr. Moestopo 47, Surabaya, Indonesia. *; 2 *Department of Pathological Anatomy, Faculty of Medicine, Airlangga University, Jalan Mayjen Prof. Dr. Moestopo 47, Surabaya, Indonesia. *; 3 *Department of Pharmacology, Faculty of Medicine, Airlangga University, Jalan Mayjen Prof. Dr. Moestopo 47, Surabaya, Indonesia. *; 4 *Department of Health Faculty of Vocational Study, Airlangga University, Jalan Darmawangsa dalam 68-69 601551 Surabaya, Indonesia. *

**Keywords:** Apoptosis, caspase-12, cisplatin, glomerular epithelial cells, glutamine

## Abstract

**Objective:**

Cisplatin is potent chemotherapy for broad-spectrum malignancies treatment, but its use is limited by organ toxicity effects, including nephrotoxicity. Glutamine prevents cisplatin nephrotoxicity by inhibiting the oxidative stress in kidney cell apoptosis.

**Methods:**

This research examined the nephroprotective effects of intravenous glutamine on the glomerular epithelium of male rats (Rattus norvegicus). 30 male rats were randomly divided into (1) P0 as the control group; (2) P1 that was administered with single dose cisplatin (20 mg/kg BW) intraperitoneal injection; and (3) P2 that was administered with intravenous injection of glutamine (100 mg/kg BW) and single-dose cisplatin (20 mg/kg BW) intraperitoneal injection. The measurement of *caspase-12* expression and apoptotic cells was performed using immunohistochemical methods.

**Results:**

The *caspase-12* expression are as follows: P0 = 0.5 ± 0.15; P1 = 4.1 ± 0.86; P2 = 2.54 ± 0.72. The apoptotic cells are as follows: P0 = 14.5 ± 5.23 cells/field of view; P1 = 52.7 ± 17.06 cells/field of view; P2 = 31.5 ± 6.73 cells/field of view. There is a decrease in the *caspase-12* expression and apoptotic cells after intravenous glutamine administration in male white rats’ glomerular epithelial cells exposed to cisplatin. The decrease of *caspase-12* expression is followed by a decrease in glomerular epithelium apoptosis after intravenous glutamine administration.

**Conclusion:**

Immunohistochemical examination can be used as a marker of the nephrotoxic effect of cisplatin on the renal glomerular epithelium. Glutamine has been observed to give nephroprotective effect to cisplatin nephrotoxic effects.

## Introduction

The prevalence of metabolic diseases in Indonesia, especially kidney disease, exhibits a high trend. Based on the 2017 data from the Health Insurance Administering Agency (BPJS), the state covered the 3,657,691 recorded cases of chronic kidney disease dialysis procedures with total cost for coverage of IDR 3.1 trillion. One of the etiologies in kidney failure is the permanent damage to the nephrons by toxins.

Cisplatin is a potent chemotherapy drug that is widely used for broad-spectrum therapy for malignancies, including head and neck, esophageal, bladder, testicular, ovarian, breast, and non-small-cell lung cancers (Fillipski et al., 2008; Reck, et al., 2010). However, the clinical application of cisplatin is limited due to the high organ toxicity effects, such as nephrotoxic, neurotoxic, ototoxic, and hepatotoxic effects (Karasawa, and Steyger, 2015; Guo et al., 2018). Cisplatin causes apoptosis at lower doses (10–100 µM) and necrotic cell death at higher doses (200–800 µM) (Hanigan and Devarajan, 2003) More than 70% of pediatric patients who received cisplatin experience kidney dysfunction (Skinner et al., 1998). Moreover, acute kidney injury occurs in 20–30% of all administered patients (Miller et al., 2010). The renal dysfunction usually begins a few days after the standard dose of cisplatin (50–120 mg/m^2^) administration and is expressed by the increasing levels of serum creatinine and blood urea nitrogen (Akcay et al., 2010). Cisplatin treatment induces a massive tubular dilation and cast formation in several tubule lumens. Focal tubular necrosis, base membrane thickening of kidney tubules and Bowman’s capsule, and focal mononuclear cell infiltration are occasionally observed as well. Renal tubular damage also occurs, together with vacuolization and desquamation of epithelial cells. Some glomeruli experience necrosis and normal residual glomeruli often show the enlarged periglomerular spaces. 

One of the cisplatin’s nephrotoxic mechanisms is apoptosis, which is a programmed cell death pathway activated by cisplatin through some mechanisms. Cisplatin decreases *Bcl-2* expression in the mitochondrial pathway, which supposedly inhibits the development of the pro-apoptotic molecules (Oltvai and Korsmeyer, 1994). In the death receptor pathways, cisplatin activates Fas-dependent pathways and apoptosis, either in vivo (Nogae et al., 1998) or in vitro (Feldenberg et al., 1999). Cisplatin also activates caspase pathways. The role of caspase-3 is first encountered in the cultured tubular proximal cells, which respond to cisplatin by increasing the caspase-3 activity in a dose-dependent manner (Fukuoka et al., 1998; Lau, 1999). Following studies have revealed caspase-9 activation and, to a lesser extent, caspase-8 activation (Kaushal et al., 2001; Park et al., 2001). 

Glutamine (Gln or Q) is an α-amino acid used in protein biosynthesis. It is one of the ingredients to produce glutathione, which is a potent antioxidant. A single dose of intravenous glutamine (0.15–0.75 g/kg) can increase the *HSP * expression, reduce end-organ injury, and improve the survival rate from septic shock in mice (Wischmeyer, 2002). Glutamine also increased the vascular reactivity by inducing *HSP* expression and inhibiting the release of inflammatory cytokines and peroxide biosynthesis in LPS shock mice (Jing et al., 2017). Glutamine can also reduce apoptosis by inhibiting the oxidative stress, as observed by the results of the trials using mice with colitis induced by a 2,4,6-trinitrobenzene sulfonic acid (Crespo et al., 2012).

The levels of caspase-12 in the glomerular epithelium have not been studied, even though the time of exposure to the toxin received to the glomerulus is high and the epithelial cells are exposed first then the tubules. A study has shown that Bowman’s capsules have a role in the physiological function of the normal glomerulus. The podocyte injury stimulates the activation of Bowman’s capsule parietal cells, which extend into the glomerular beam, resulting in segmental and global sclerosis by producing more matrixes and destroying the glomerulus capillary’s lumen (Al Hussain et al., 2017).

This study aims to analyze the nephroprotective effect of intravenous glutamine on the glomerular epithelial cells apoptosis by examining the expression of *caspase-12*, which is the initiator of apoptosis in the endoplasmic reticulum stress pathway. Pro-caspase-12 is found in the endoplasmic reticulum, especially in the proximal tubules of the kidney. The intravenous glutamine is expected to be an alternative solution for kidney failure caused by cisplatin chemotherapy modalities.

## Materials and Methods

*Animals and Treatment*

This research was approved by the Health Research Ethics Committee of the Universitas Airlangga School of Medicine (No. 25/EC/KEPK/FKUA/2020). In this research, 30 male white rats (Rattus norvegicus), aged 2–3 months with a bodyweight of 150–200 grams were obtained from the experimental animal unit of the Faculty of Veterinary Medicine, Universitas Airlangga. Each of them was held separately in a plastic box, lined with husks in the bottom, and then covered with woven wires on top. The husks were replaced every day. 

*Chemicals*

The glutamine was obtained from Serva (Germany). The glutamine solution was prepared in 0.9% NaCl, with a dose of 1 g of glutamine in 10 mL of 0.9% NaCl. The glutamine dose is injected 100 mg/kg of body weight (BW) and a single injection is made a maximum of 0.2 mL IV . The glutamine was injected intravenously into the rat’s tail using a 1 mL 27-gauge syringe.

The cisplatin material was obtained from Kalbe Farma (Indonesia), with a dose of 20 mg/kg BW, administered via intraperitoneal injection using a 1 mL 27-gauge syringe. We also used Primary Antibodies Caspase-12 (Polyclonal Antibody PA5-27094 Thermo Scientific) and Apoptosis Detection Kit in Situ Cell POD (11684817910 ROCHE).

*Experimental Design*

In this research, 30 Wistar male white rats were randomly divided into three groups at the start of the study. The control group (P0) was sacrificed by cervical dislocation after anesthesia with ether and then the kidneys were taken for immunohistochemistry preparation.

On day 7, the P1 group was administered with cisplatin at a single dose of 20 mg/kg BW via intraperitoneal injection and then observed for 72 h. The rats were sacrificed on the 10th day by cervical dislocation after anesthesia with ether, and then the kidneys were taken for the immunohistochemistry preparation. The execution time is due to the glomerular epithelial cells’ apoptosis is occurred after cisplatin injection on day 10 (Tsuruya et al., 2003). The P2 group was administered with glutamine at a dose of 100 mg/kg BW once daily via intravenous injection from day 1 to day 7 (Zhang et al., 2009). On day 7, the rats were administered with cisplatin at a single dose of 20 mg/kg BW via intraperitoneal injection. Subsequently, the rats were observed for 72 h and then sacrificed on the 10th day through cervical dislocation after anesthesia with ether. Later, the kidneys were taken as well for the immunohistochemistry preparation. 

*Histopathological Preparation*

The kidney tissue was fixed in 10% formalin for 15–24 h. The dehydration was performed for 60 minutes using the series of alcohol (30%, 50%, 70%, 80%, 96%, and absolute concentration) to prevent any changes in tissue morphology. Then, the clearing using xylol was performed twice (60 min each). Subsequently, the infiltration with tender paraffin was performed for 60 min at 48°C. Later, the hard paraffin was blocked in the mold and stored for 1 day. The next day, the hardened was placed on the holder and cut into 4–5-μm-thick sections using a rotary microtome. After that, the mounting to object glass was performed with the poly-L-lysine coating. The mounted tissue was immersed in xylol twice (5 min each). After that, the rehydration was performed using the series of alcohol (absolute, 96%, 80%, 70%, 50%, and 30% concentration) for 5 min, respectively. Then, it was rinsed in dH_2_O for 5 min.

*Immunohistochemical Assay of CSP12*

The slides were washed once with phosphate-buffered saline (PBS) (Millipore 524650) at pH 7.4 for 5 min three times. The endogenous peroxide blocking was carried using 3% H2O2 for 20 min. Then the slides were washed again with the same procedure before the blocking treatment. The unspecific protein blocking was done using 5% PBS (Millipore 524650) containing 0.25% Triton X-100 (Sigma-Aldrich T8787) and then repeat the washing procedure. The incubation of primary antibodies caspase-12 (Polyclonal Antibody PA5-27094 Thermo Scientific) was done overnight at 40°C. After that, the slides were washed using PBS (Millipore 524650), pH 7.4, three times for 5 min each. The incubation using anti-mouse biotin-conjugated was done for 1 h at room temperature then the wash procedure was repeated. The incubation using streptavidin-horse radish peroxidase (Thermo Fisher Scientific N100) was done for 40 min and then washed once more. The slides were then added with drops with diaminobenzidine (DAB) (Thermo Fisher Scientific 3400) and incubated for 10 min and then washed with a similar procedure. The counterstaining was using Mayer’s hematoxylin solution (Sigma-Aldrich MHS32) for 10 min, then washed using tap water, rinsed with dH2O, and air-dried. The prepared slides were observed using a light microscope at a magnification of 400× after mounted and covered with a glass cover. The positive molecular expression with primary antibodies would appear brown when observed. The calculated glomerular epithelial cells were located in the renal corpuscle area marked by a flat layer of epithelial cells at the parietal part of Bowman’s space.

*Apoptosis Examination*

Tissue deparaffinization was performed by washing the tissue three times with xylene (Supelco 108297) for 5 min. The tissue was soaked twice in absolute ethanol for 5 min each and then transferred to 95% ethanol and 70% ethanol 3 min each. The tissue was washed with PBS (Millipore 524650) for 5 min.

The antigen uptake was done by giving proteinase K (Sigma-Aldrich 70663) for 15 min to the sample. Then, the tissue was washed twice with H_2_O in a Coplin tube for 2 min. The endogenous peroxidase was removed by dripping 3% H_2_O_2_ PBS and left for 5 min at room temperature. Then, the tissue was washed twice with PBS (Millipore 524650) for 2 min each in a Coplin tube. The excess liquid was dried using paper. About 75 μl of equilibration buffer was dropped on the tissue and then incubated for 10 min at room temperature. The liquid around the tissue was then dried using paper. The enzyme activity was reduced by 55 μl/5 cm in the tissue of TdT (Sigma-Aldrich SAB5600268), and then the tissue was incubated at 37°C in a moist container for 1 h. The preparations were placed in a Coplin jar containing a strong buffer/cleaning job and then incubated for 10 min at room temperature. The preparations were washed four times with PBS (Millipore 524650) for 2 min in a Coplin jar at room temperature. The excess liquid around the tissue was dried using paper. Anti-digoxigenin (Roche 11333089001) conjugate, which was previously removed from storage and warmed to room temperature, was dropped on a tissue surface of 65 μl/5 cm^2^ and then incubated for 30 min at room temperature. The preparation was washed four times with PBS (Millipore 524650) in a Coplin jar for 2 min at room temperature. Then, the excess liquid around the tissue was dried using paper. The color was exposed after peroxidase substrate (Sigma-Aldrich CPS160) drops of 75 μl/5cm2 on the surface of the tissue and left for 10 min at room temperature. The preparations were washed three times with dH_2_O for 1 min in Coplin jars and then incubated with dH_2_O in Coplin jars for 5 min at room temperature. The methyl green counterstaining (Sigma-Aldrich M8884) was performed for 30 s at room temperature. Then, the preparation was washed three times with dH_2_O for 1 min in a Coplin jar. The preparations were cleaned with xylene (Supelco 108297), and the excess liquid around the tissue was dried using paper. The preparations were observed using a light microscope at a magnification of 400× and the brown epithelial cells from the glomerular kidney epithelium were counted in each field of view. 

*Statistical Analysis*

The ratio was the used data scale for caspase-12 expressing cells and apoptotic cells, so it was necessary to conduct a normality test using the Shapiro–Wilk test (α = 0.05) and homogeneity test using the Leaven test (α = 0.05). If the results were in the normal distribution and homogeneous, then a different test using one-way ANOVA (α = 0.05) was conducted. If the results were normal but heterogeneous, then the conducted test was Kruskal–Wallis (α = 0.05). If there was a difference in data obtained, then the analysis was followed by Mann–Whitney U test (α = 0.05). Eventually, Pearson’s correlation test was conducted to find out the correlation between caspase-12 expressing cells and apoptotic cells.

## Results

*Glomerular Epithelial Cells Morphology*

Before observing the caspase-12 expressing cells and apoptotic cells, the morphology of Bowman’s capsule renal epithelium was first observed with hematoxylin–eosin (HE) staining at a 400× magnification. The observed object was chosen randomly to represent the histological picture of each group. The cisplatin was occasionally found in focal tubular necrosis, the thickening of the base membrane in the kidney tubules and Bowman’s capsules, and the infiltration of focal mononuclear cells in the glomerulus. Some glomeruli experience necrosis and normal residual glomeruli often exhibit enlarged periglomerular spaces. 

*Caspase-12 Protein Expression and Apoptotic Cells in the Glomerular Epithelial Cells*

The results of immunohistochemical-stained kidney tissue exhibited changes in the expression of *caspase-12* in each group. The administration of cisplatin injection in group P1 exhibited a higher mean expression of *caspase-12* after 72 h (4.1 ± 0.86) when compared with the control group (P0). The administration of glutamine injection and cisplatin injection (P2) exhibited a lower mean expression of *caspase-12* after 72 h (2.54 ± 0.72) when compared with P1 (Table 1).

Besides, cisplatin injection in group P1 exhibited a higher rate of cell apoptosis after 72 h (52.7 ± 17.06) when compared with the control group (P0). The administration of glutamine injection and cisplatin injection in P2 exhibited a lower mean expression of *caspase-12* after 72 h (31.5 ± 6.73) when compared with P1 (Table 1).

The Kruskal–Wallis test indicated the differences in the number of glomerular epithelial cells expressing caspase-12 and in the number of apoptotic cells in the three treatment groups. Subsequently, the Mann–Whitney U test concluded that there were differences in the number of parietal epithelial cells expressing caspase-12 protein and in the number of apoptotic cells between the P1 and P2 groups with the control group and between the P1 and P2 groups. There were also differences in the number of parietal epithelial cells expressing the caspase-12 protein and the number of cell apoptosis (Table 2).

*The Correlation Between Capase-12 Expression Cells and Apoptotic Cells*

The Pearson’s correlation test results indicated that a significant correlation exists between the glomerular epithelial cells expressing caspase-12 and the number of cells undergoing apoptosis with the correlation coefficient of 0.707, which is strong and positive. Thus, it can be concluded that if the number of protein expression cells decreases, the number of apoptotic cells also decreases, and vice versa.

**Table 1:** The Descriptive Statistic of Caspase-12 Expression and Apoptotic Cells. P0: control group. P1: cisplatin injection on the 7^th^ day. P2 treated with intravenous glutamine injection 7 days in a row before being injected with cisplatin on the 7^th^ day

Group	Cells expressing the caspase-12 protein Mean ± SD	Apoptotic cells Mean ± SD
P0	0.5 ± 0.15	14.5 ± 5.23
P1	4.1 ± 0.86	52.7 ± 17.06
P2	2.54 ± 0.72	31.5 ± 6.73

**Figure 1:**
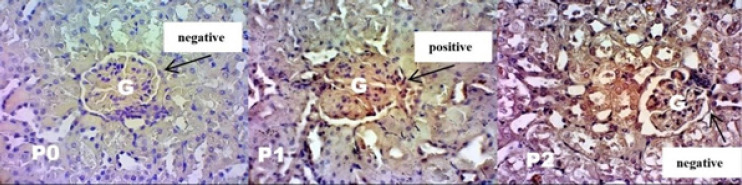
Glomerular Epithelial Cells Morphology after CSP-12 Antibody. P0: control group. P1: cisplatin injection on the 7^th^ day. P2 treated with intravenous glutamine injection for 7 days in a row before being injected with cisplatin on the 7^th^ day. The positive result indicated with brown cytoplasm

**Figure 2:**
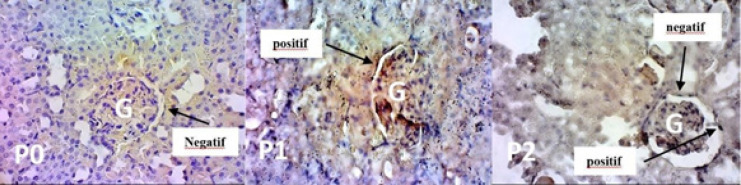
Morphology of the Glomerular Epithelial Cells after Apoptosis Detection Kit. P0: control group. P1: cisplatin injection on the 7^th^ day. P2 treated with intravenous glutamine injection for 7 days in a row before being injected with cisplatin on the 7^th^ day. The positive result indicated with brown cytoplasm

**Table 2:** Mann–Whitney U Test Results

Variable	Comparison		P-value	Interpretation
Cells expressing the caspase-12	P0	P1	0	Obtained difference
		P2	0	
	P1	P2	0.001	
Apoptotic cells	P0	P1	0	Obtained difference
		P2	0	
	P1	P2	0.001	

## Discussion

In this study, the treatment was performed in the experimental animals that were divided into three groups: the control group (P0); the P1 group, which received intraperitoneal cisplatin injection; and the P2 group, which received intraperitoneal cisplatin injection and intravenous glutamine injection. The treatment response was observed for 72 h after treatment because apoptosis was apparent after 72 h of cisplatin injection (Tsuruya et al., 2003). The site observed in this study was the glomerular epithelium. So far, there has been no research yet on the cisplatin nephrotoxicity in the glomerular epithelium. The glomerulus is more proximal than the tubules and therefore is thought to be affected by cisplatin nephrotoxicity earlier than the tubules.

 The differences in the *caspase-12* expression level were directly proportional to the level of glutamine administration in each treatment. These results are supporting by a study that stated that glutamine can induce high levels of *HSP* expression in all organs, including the kidneys (Zhang et al., 2009) A single dose of intravenous glutamine can increase the *HSP* expression, reduce end-organ injury, and improve survival from septic shock in whole mice (Wischmeyer, 2002). The cytoprotective and antioxidant properties of glutamine may be crucial in high catabolism situations, where the activity and expression of inflammatory pathways mediated by NF-κB are modulated (Cruzat et al., 2014). The decreased availability of plasma glutamine has been reported to cause the reduction of lymphocyte proliferation, impair the expression of surface activation proteins and cytokine production, and induce apoptosis in cells (Roth, 2008). This is what might be the reason glutamine can prevent the cells toward apoptosis in this study.

As mentioned in the results, glutamine administration decreased apoptosis of cisplatin-treated cells. This has been proven to the contrary in a study by Kadri et al. (2017) where the suppression of glutamine metabolism can increase the cytotoxic potential of some therapeutic drugs, such as cisplatin, thus allowing a decrease of chemotherapy compounds dose to minimize the toxicity and adverse reactions.

The mechanisms of cell death depend on the cisplatin administration dose, either by necrosis or apoptosis (Lieberthal et al., 1996). In in vitro experiments, low concentrations of cisplatin cause cells to undergo apoptosis, while necrosis at high concentrations (Megyesi et al., 1998; Ramesh and Reeves, 2003). In this research, the dose was enough to cause the epithelial cells to undergo apoptosis. 

In the present study, cisplatin injection in the P1 exhibited a higher rate of cell apoptosis after 72 h compared to the control (P0). We also found that glutamine and cisplatin injection in the P2 expressed lower *caspase-12* expression after 72 h compared to P1.

Apoptosis after cisplatin treatment can also involve the ER -stress pathway. The caspase initiator in the ER pathway is caspase-12, which is localized to the cytosolic face of the ER and activated by ER pressure (Boyce and Yuan, 2006). Caspase-12 is activated during the treatment of cisplatin in LLC-PK1 cells (Liu and Baliga, 2005). In vitro observations have recently been extended to mouse models of cisplatin nephrotoxicity, where ER stresses and associated signaling, such as cleavage of caspase-12, were observed (Peyrou et al., 2007).

It can be concluded that apoptosis due to cisplatin administration occurs due to the increased expression of *caspase-12*, which induces apoptosis in the renal glomerular epithelial cells. At 72 h of observation after intravenous glutamine administration, the incidence of apoptosis in the renal glomerular epithelial cells was significantly inhibited (p < 0.0001) between the groups and was directly proportional to the effect of glutamine, which inhibited the expression of *caspase-12*.

In conclusion, based on the results, there is a decrease in the expression of *caspase-12* and apoptotic cells in the glomerular epithelial cells of male white rats exposed to cisplatin after intravenous glutamine administration. The decreased expression of *caspase-12* in intravenous glutamine administration is followed by a decrease in apoptosis in the glomerular epithelium. Thus, it is assumed that immunohistochemical examination on the renal glomerular epithelium can be used as a marker for the nephrotoxic effect of cisplatin.

The limitation of this study is various variables have not been observed. The researcher did not make observations to the executor caspase (caspase-3) because the researcher wanted to see the significance if the treatment was carried out in the initiator caspase. This study also did not see the incidence of apoptosis through two or more routes. In this case, the researcher wants to try to go through one route first and there is no need to study more than one route if the results are significant. It is also suggested to do further research on the application of glutamine intravenous to humans receiving cisplatin chemotherapy and also on the treatment in the animal with cancer so the effect of glutamine in cancer group and non-cancer can be observed.
